# Small RNA mobility and plant virus diseases

**DOI:** 10.1093/jxb/eraf226

**Published:** 2025-05-21

**Authors:** Laura Elvira-González, Todd Blevins, Manfred Heinlein

**Affiliations:** Institut de Biologie Moléculaire des Plantes, CNRS, Université de Strasbourg, Strasbourg 67084, France; Institut de Biologie Moléculaire des Plantes, CNRS, Université de Strasbourg, Strasbourg 67084, France; Institut de Biologie Moléculaire des Plantes, CNRS, Université de Strasbourg, Strasbourg 67084, France; University of Wisconsin–Madison, USA

**Keywords:** Disease, microRNAs, plants, plasmodesmata, RNA silencing, small interfering RNAs, tolerance, viral suppressor of RNA silencing, VSR, viruses

## Abstract

Plants rely on symplasmic networks of cell-to-cell communication through plasmodesmata and long-distance communication through phloem to regulate plant development and adaptations to environmental changes. Plasmodesmata facilitate the intercellular transport of metabolites, phytohormones, proteins and RNA molecules, many of which act as signaling molecules. Among these, non-cell-autonomous RNA molecules play a crucial role in coordinating plant development, gene silencing, stress responses, and nutrient allocation, as well as in antiviral defense and host–parasite interactions. This review explores the mechanisms of cell-to-cell and systemic mobility of small RNAs, with a particular emphasis on the role of virus- and host-derived small RNAs in regulating the outcome of viral infection in terms of disease, resistance and tolerance.

## Teaser

This review describes how virus-induced small RNA synthesis and small RNA movement through plasmodesmata and phloem determine the outcome of viral infection in terms of disease and tolerance.

## Introduction

**Fig. 1.**
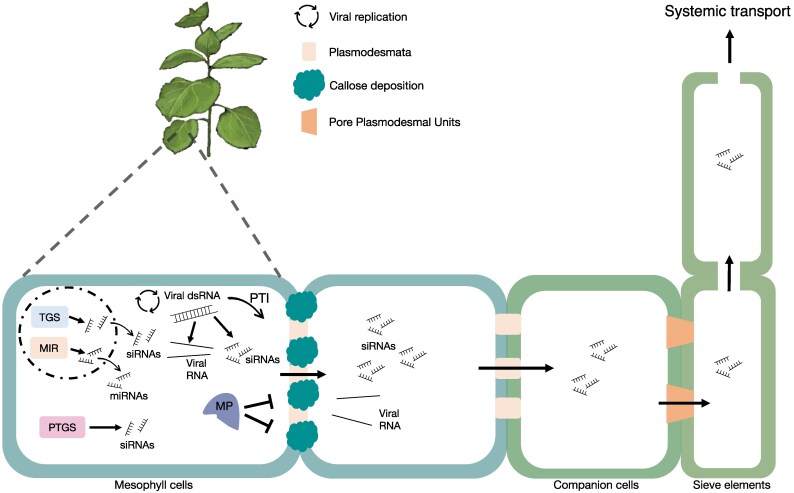
Dynamic regulation and role of plasmodesmata (PD) in viral RNA and siRNA movement. PD regulate the symplasmic transport of molecules between cells. Viruses encode movement proteins (MPs) that are expressed in cells at the viral infection front to facilitate viral movement through PD. Tobamovirus MPs interfere with pattern-triggered immunity (PTI), a defense mechanism activated by plant receptors that recognize viral double-stranded RNA (dsRNA) and cause PD closure by callose deposition. The open state of the PD at the viral infection front, which allows the virus to spread, also allows the movement of signaling molecules, including viral and host-derived small interfering RNAs (siRNAs). As they move ahead of the infection, these siRNAs play a critical role in systemic defense signaling and the regulation of disease outcomes. MIR, miRNA-encoding gene; PTGS, post-transcriptional gene silencing; TGS, transcriptional gene silencing.

**Fig. 2.**
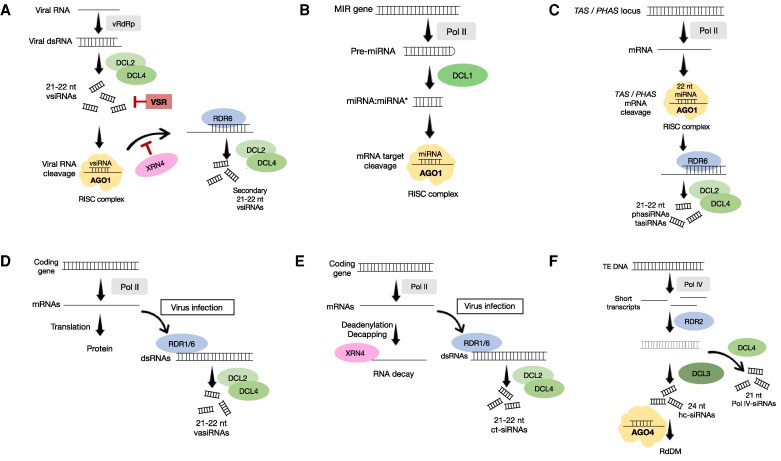
Overview of small RNA biogenesis pathways. (A) Biogenesis of virus-derived small interfering RNAs (vsiRNAs) and their role in antiviral RNA silencing. Virus replication involves the production of double-stranded RNA (dsRNA), which is processed by plant DICER-LIKE (DCL) 4 and DCL2 enzymes into 21 nt and 22 nt vsiRNA duplexes, respectively. One of the strands of the duplex associates with ARGONAUTE1 (AGO1) to direct sequence-specific cleavage of the viral RNA by the AGO1-containing RNA-induced silencing complex (RISC). The cleavage product is used as a template for new dsRNA synthesis by RNA-DEPENDENT RNA POLYMERASE 6 (RDR6). This dsRNA is again cleaved by DCL2/4, leading to the accumulation of secondary vsiRNAs that amplify the antiviral RNA silencing response. RNA decay pathway enzymes (e.g. the 5′–3′ exoribonuclease XRN4) can inhibit secondary vsiRNA production through substrate competition with RDR6. Viral suppressors of RNA silencing (VSRs) inhibit the antiviral RNA silencing pathway by siRNA sequestration (as shown) or by other mechanisms. (B) Production of microRNAs (miRNAs) and their function in post-transcriptional gene silencing. miRNA-encoding *MIR* genes are transcribed by RNA polymerase II (Pol II) into precursor miRNAs (pre-miRNAs), which are processed by DCL1 into miRNA:miRNA* duplexes. The miRNA directs RISC to target mRNAs for sequence-specific cleavage or translational repression. (C) Biogenesis of phased small interfering RNAs (phasiRNAs) and *trans*-acting siRNAs (tasiRNAs). *TAS*/*PHAS* loci are transcribed by Pol II, and tasiRNAs/phasiRNAs are generated through initial cleavage of the transcript guided by 22 nt miRNAs followed by conversion of the cleaved transcript into dsRNA by RDR6 and subsequent processing by DCL2/4. (D) Biogenesis of virus-activated vasiRNAs (vasiRNAs). Virus infection triggers the RDR1/6-dependent conversion of mRNAs derived from coding genes into dsRNAs, which are processed by DCL2/4 into vasiRNAs. vasiRNA production controls the transcript levels and translation of coding genes. (E) RNA decay and production of coding transcript-derived siRNAs (ct-siRNAs). RNA decay plays an important role in regulating mRNA levels and degrading aberrant RNA molecules. After decapping and deadenylation, these RNAs are degraded by exonucleases such as XRN4 and the exosome. The RNA decay pathway can be overwhelmed by viral RNA that accumulates during infection. This leads to RNA degradation by post-transcriptional RNA silencing, including the conversion of mRNAs into dsRNA by RDR1/6 and subsequent processing into ct-siRNAs by DCL2/4. (F) Production of heterochromatic siRNAs (hc-siRNAs) and Pol IV-dependent 21 nt siRNAs. Transposable element (TE) regions in the nucleus are transcribed by RNA polymerase IV (Pol IV) and the resulting short transcripts are converted into dsRNAs by RDR2. These dsRNAs are primarily processed by DCL3 to generate hc-siRNAs, which guide RNA-directed DNA methylation (RdDM) via AGO4. The dsRNAs can also be processed by DCL4 to generate 21 nt Pol IV-dependent siRNAs (Pol IV-21-siRNAs).

Intercellular communication is crucial for plant development, disease resistance, and responses to environmental stimuli. A large part of this communication occurs through plasmodesmata (PD), the gated plasma membrane-lined cytoplasmic nanochannels in plant cell walls. Together with the connected phloem, the PD establish a cytoplasmic continuum through which small molecules, including metabolites and hormones, as well as proteins and RNAs, can travel between cells, tissues, and organs (Lucas and Lee, 2004; Bayer and Benitez-Alfonso, 2024; Tee and Faulkner, 2024). To control intercellular transport, PD are equipped with signaling hubs that regulate the PD aperture, that is, the PD size exclusion limit, in response to external and internal cues. A key mechanism for regulating the aperture of PD is callose deposition in the cell wall that surrounds the PD (Levy *et al.*, 2007; Simpson *et al.*, 2009; Vaten *et al.*, 2011; Cui and Lee, 2016; Xu *et al.*, 2017; Gaudioso-Pedraza *et al.*, 2018; Huang *et al.*, 2023). Callose deposition may force the plasma membrane toward the centrally located strand of the endoplasmic reticulum (the desmotubule), thus limiting the size of the cytoplasmic space between the two membranes available for molecular transport. Viruses target the PD to spread infection from one cell to the other. To achieve movement, they temporarily adjust the PD size exclusion limit with the help of their movement proteins (MPs) (Wolf *et al.*, 1989; Oparka *et al.*, 1997; Waigmann *et al.*, 2000). Recent studies have suggested that MPs do not open the PD, but rather prevent PD closure triggered by a host immunity signaling response. This response is elicited by receptor-mediated perception of viral double-stranded RNA (dsRNA), which is generated by the viral RNA-dependent RNA polymerase and occurs as a hallmark intermediate of viral replication (Niehl *et al.*, 2016; Huang *et al.*, 2023). However, while the MPs block the closing of PD at the viral infection front to allow virus movement, they also likely enable the dissemination of other molecules from the infected cells, particularly virus-derived small interfering RNAs (vsiRNAs), which are key antiviral molecules (Baulcombe, 2022) (Fig. 1). vsiRNAs are produced upon activation of the antiviral RNA silencing pathway (Fig. 2A), in which the viral dsRNA is cleaved by DICER-LIKE (DCL) 4 and DCL2 proteins into 21 nt and 22 nt small interfering RNA (siRNA) duplexes, respectively (Gasciolli *et al.*, 2005; Xie *et al.*, 2005; Blevins *et al.*, 2006; Bouché *et al.*, 2006; Deleris *et al.*, 2006). The resulting vsiRNA duplex is then loaded on to ARGONAUTE (AGO)-associated RNA-induced silencing complexes (RISCs), with one vsiRNA strand being retained to guide the targeting of viral RNA for sequence-specific cleavage, thereby causing the inhibition of virus accumulation (Hamilton and Baulcombe, 1999; Morel *et al.*, 2002; Zhang *et al.*, 2006). The cleaved viral RNA is either degraded or serves as a template for new dsRNA synthesis by RNA-DEPENDENT RNA POLYMERASE 6 (RDR6), leading to the production of secondary vsiRNAs, which can amplify the antiviral RNA silencing response and its systemic propagation between cells (Fig. 2A) (Mourrain *et al.*, 2000; Schwach *et al.*, 2005; Wang *et al.*, 2011). In addition, RNA-DEPENDENT RNA POLYMERASE 1 (RDR1), a salicylic acid-inducible enzyme that mediates antiviral silencing responses, provides a plant stem cell-specific antiviral immunity mechanism (Yang *et al.*, 2004; Incarbone *et al.*, 2023).

To counteract silencing, viruses produce viral suppressors of RNA silencing (VSRs) that inhibit the host RNA silencing pathway at various stages, often by sequestering vsiRNAs or blocking their incorporation into RISCs (Lakatos *et al.*, 2006; Merai *et al.*, 2006; Csorba *et al.*, 2007, 2015; Vogler *et al.*, 2007; Jamous *et al.*, 2011). However, while inner cells of the viral infection site in leaves accumulate VSRs to the high levels required for the suppression of antiviral RNA silencing, cells at the infection front accumulate only low levels of VSRs. This was demonstrated, for example, by the observation that green fluorescent protein (GFP)-encoding tobacco mosaic virus (TMV) is unable to spread into cells in which RISCs have already been preloaded with GFP siRNAs targeting the viral RNA, indicating low VSR activity in cells at the infection front (Vogler *et al.*, 2007). Evidence for differential expression of VSRs between cells at and behind the infection front is also provided by tobacco plants carrying the *N* resistance gene, in which the VSR of TMV induces a hypersensitive response leading to cell death. Whereas cells behind the infection front of the local infection sites in the leaves undergo cell death, cells at the infection front survive and are able to spread the infection further (Wright *et al.*, 2000). Cells at the viral infection front are therefore in a special state in which their low VSR activity is coupled to open PD. As a likely consequence, cells can shed vsiRNAs and other small RNAs into neighboring and distant cells, thereby influencing the course of viral infection (Fig. 1).

The profiles of small RNAs that can be disseminated by cells at the infection front are expected to have a specific composition, as viral infection leads to significant changes in the small RNA profiles of plant cells (Blevins *et al.*, 2011; Du *et al.*, 2011; Hu *et al.*, 2011; Wang *et al.*, 2011; Cao *et al.*, 2014; Garcia-Ruiz *et al.*, 2015; Pitzalis *et al.*, 2020; Leonetti *et al.*, 2021). These changes include the presence of vsiRNAs, which can account for 30–40% of all small RNAs, but also host-derived microRNAs (miRNAs) and siRNAs, some of which are highly induced by viral infection. This review discusses the altered small RNA profiles of virus-infected cells and how the systemic distribution of small RNAs through the PD and phloem may regulate plant–virus compatibility and the outcomes of infection.

## Effects of virus infection on host small RNA profiles

Small RNAs are classified as miRNAs or siRNAs based on their respective mechanisms of biogenesis. miRNAs, which are usually 21–22 nt long, regulate genes involved in plant physiology, growth, and development, including those encoding transcription factors and stress-response proteins (Rogers and Chen, 2013). They arise when MIR genes are transcribed by RNA polymerase II into primary miRNAs (pri-miRNAs) that fold into imperfect stem-loop structures, which are successively processed by DCL1 into precursor miRNAs (pre-miRNAs) and then miRNA/miRNA* duplexes of which the miRNA strand then guides AGO-RISC to target mRNAs for cleavage or translational inhibitione (Fig. 2B). Some pre-miRNAs form longer hairpins encoding multiple miRNAs (Zhang *et al.*, 2010; Rosatti *et al.*, 2024). Studies with different viruses, including oilseed rape mosaic virus (ORMV) or rice stripe virus, have shown that in addition to canonical miRNAs present in healthy plants, infected plants accumulate phased miRNA/miRNA* sequences derived from such long hairpins (Du *et al.*, 2011; Hu *et al.*, 2011). In addition, viral infections can generally alter miRNA abundance and function (Xia *et al.*, 2018; J. Liu *et al.*, 2020; Pitzalis *et al.*, 2020). Although such changes in miRNA levels have been correlated with changes in target mRNA levels in cells at the virus infection front (e.g. Pitzalis *et al.*, 2020), such correlations were not observed in Arabidopsis plants already systemically infected with ORMV, in which miRNA activity may have been inhibited by sequestration by the VSR (Hu *et al.*, 2011).

Virus infection also alters the profile of host-derived siRNAs. Unlike miRNAs, siRNAs are derived from long RNA transcripts that in certain circumstances are converted to dsRNAs and cleaved into siRNAs. The first identified endogenous siRNAs were *trans*-acting siRNAs (tasiRNAs) that are derived from non-coding *TAS* gene loci and are conserved in terrestrial plants (Vazquez *et al.*, 2004; Allen *et al.*, 2005; Axtell *et al.*, 2006; Y. Liu *et al.*, 2020). tasiRNA production begins with miRNA-guided cleavage of the precursor RNA, followed by double-strand formation via RDR6. The miRNA-guided cleavage sets the phasing register for subsequent DCL processing of the dsRNA, resulting in the production of phased siRNAs, or ‘phasiRNAs’, that align precisely when mapped to the precursor RNA (Allen *et al.*, 2005; Axtell *et al.*, 2006) (Fig. 2C). The miRNAs that direct the initial cleavage are typically 22 nt long. In addition, DCL2-dependent 22 nt siRNAs can initiate cleavage of the mRNA (Chen *et al.*, 2010). The processing of phasiRNAs is referred to as ‘transitive’ because the resulting siRNAs differ in sequence and map downstream of the miRNA or siRNA cleavage sites that initiated this process. In addition to *TAS* genes, several other secondary phasiRNA-producing loci (commonly referred to as *PHAS* loci) have been identified in several species, and these loci can be both protein-coding and non-coding (Fei *et al.*, 2013; Y. Liu *et al.*, 2020). These phasiRNAs can act *in trans* to target genes involved in processes such as abiotic stress tolerance, wounding, parasitism, seed germination, flowering, and premeiotic development (Y. Liu *et al.*, 2020).

Viral infection can have inhibitory and strong stimulatory effects on phasiRNA synthesis. An important example are phasiRNAs derived from genes for nucleotide-binding leucine-rich repeat (NLR) immune receptor proteins, which are critical for effector-triggered immunity (ETI) during viral infection. Because constitutive NLR expression and defense gene activation has a fitness cost and can trigger a hypersensitive response leading to cell death, their expression is controlled by miRNAs from the miR482/2118 superfamily (Li *et al.*, 2012; Shivaprasad *et al.*, 2012; Fei *et al.*, 2015; Xia *et al.*, 2015). Under normal conditions, these miRNAs maintain low NLR levels by converting NLR transcripts to phasiRNAs. However, viruses encoding VSRs that sequester small RNAs, such as tobamoviruses and tombusviruses (Csorba *et al.*, 2015), can sequester the initiator miRNA, which may lead to the inactivation of phasiRNA production from resistance genes, including NLRs against viral effector proteins, thereby triggering ETI (Fei *et al.*, 2016). However, cells at the infection front with only low VSR activity may not be able to achieve this inhibition and may instead disseminate *NLR*-derived siRNAs to keep *NLR* expression at low levels in neighboring cells, thereby increasing tissue compatibility for the spreading virus.

Unlike phasiRNAs derived from *NLR* genes, which can be repressed during viral infection, other phasiRNAs are highly induced by infection. Arabidopsis and other Brassicaceae infected with turnip mosaic virus, cucumber mosaic virus, or cauliflower mosaic virus accumulate substantial amounts of 21 nt and 22 nt siRNAs, termed ‘virus-activated siRNAs’ (vasiRNAs) (Cao *et al.*, 2014; Pitzalis *et al.*, 2020; Leonetti *et al.*, 2021) (Fig. 2D). Like the phasiRNAs derived from *NLR* genes, vasiRNAs are derived from mRNAs transcribed from coding genes, which are converted to dsRNA by either RDR1 or RDR6 followed by DCL cleavage (Cao *et al.*, 2014). The processing of mRNAs into vasiRNAs during viral infection may allow plants to control mRNA levels and translation not only of the gene of origin but also of other homologous genes (i.e. other members of the same gene family). In addition, several vasiRNAs have been shown to potentially target more distantly related genes for mRNA cleavage during infection (Cao *et al.*, 2014; Pitzalis *et al.*, 2020; Leonetti *et al.*, 2021).

Virus-infected cells also have altered small RNA profiles due to the viral inhibition of RNA decay, a process that removes redundant or aberrant mRNAs. Normally, mRNAs are degraded by the RNA exosome complex (3′–5′) or by the exonuclease XRN4 (5′–3′) (Zhang and Guo, 2017). Viral RNA accumulation can overwhelm RNA decay pathways, preventing the degradation of aberrant mRNAs. This perturbation can lead to post-transcriptional gene silencing, with transcripts being converted into dsRNA by RDR6 and processed into ‘coding transcript-derived siRNAs’ (ct-siRNAs) by DCL2 and DCL4 (Gazzani *et al.*, 2004; Moreno *et al.*, 2013; Zhang *et al.*, 2015; Feng *et al.*, 2024) (Fig. 2E). Similar to vasiRNAs, ct-siRNAs are derived from coding genes. Further studies may reveal whether vasiRNAs and ct-siRNAs are separate or overlapping siRNA species in infected cells.

However, viral infection not only interferes with plant genome-derived siRNAs in the cytoplasm (e.g. tasiRNAs, phasiRNAs, vasiRNAs, and ct-siRNAs) but also affects the accumulation of ‘heterochromatic siRNAs’ (hc-siRNAs) in the nucleus. These 24 nt siRNAs mostly derive from plant transposable elements and mediate RNA-directed DNA methylation (RdDM) and transcriptional gene silencing (TGS) (Fig. 2F). hc-siRNA biogenesis is initiated by RNA polymerase IV (Pol IV) (Herr *et al.*, 2005; Onodera *et al.*, 2005; Rymen *et al.*, 2020), which transcribes chromosomal DNA into short 26–45 nt transcripts (Blevins *et al.*, 2015; Zhai *et al.*, 2015), which are then directly channeled into the physically associated enzyme RNA-DEPENDENT RNA POLYMERASE 2 (RDR2) for the synthesis of short dsRNAs (Singh *et al.*, 2019; Huang *et al.*, 2021). These dsRNAs are diced by DCL3 into 24/23 nt siRNA duplexes (Xie *et al.*, 2004; Blevins *et al.*, 2015; Loffer *et al.*, 2022). The 24 nt siRNA strand then guides ARGONAUTE 4 (AGO4) to nascent transcripts of RNA polymerase V, recruiting DOMAINS REARRANGED METHYLASE 2 to mediate RdDM (Zilberman *et al.*, 2003; Wierzbicki *et al.*, 2008). Importantly, in the absence of DCL3, Pol IV–RDR2-derived dsRNAs can be processed by DCL4 into 21 nt siRNAs (Gasciolli *et al.*, 2005; Henderson *et al.*, 2006; Smith *et al.*, 2007; Panda *et al.*, 2020). While the 24 nt siRNAs derived from the canonical Pol IV–RDR2–DCL3 pathway have been suggested to target the genomes of DNA viruses for RdDM (Vanitharani *et al.*, 2005; Raja *et al.*, 2008; Rodriguez-Negrete *et al.*, 2009; Wang *et al.*, 2018; Mei *et al.*, 2020; Omae *et al.*, 2020), the 21 nt siRNAs produced by alternative dicing of Pol IV–RDR2 products by the DCL4 pathway may have different functions. For example, together with vsiRNAs, vasiRNAs, and ct-siRNAs, they could contribute to a virus-induced, systemically mobile siRNA pool that may play an important role in controlling disease symptoms during viral infection, as discussed further below.

## Small RNAs are mobile

The mobility of small RNAs was indicated by experiments suggesting that they can function as intercellular gene-silencing signals. Grafting experiments demonstrated that silencing of a transgene in the rootstock can be transferred to the scion expressing a non-silenced version of the gene (Palauqui *et al.*, 1997). Systemic RNA silencing was also observed in GFP-transgenic plants after localized GFP overexpression (Voinnet and Baulcombe, 1997), and was later found to depend on RDR6, indicating the role of secondary siRNA amplification (Himber *et al.*, 2003; Schwach *et al.*, 2005). Genetic screens in silencing reporter plants identified additional factors involved in siRNA synthesis and cell-to-cell silencing, including DCL1, DCL4, HEN1, AGO1, CLSY1, Pol IV, and RDR2 (Dunoyer *et al.*, 2007; Smith *et al.*, 2007). In the canonical RdDM pathway (reviewed in Rymen *et al.*, 2020), CLSY1, Pol IV, and RDR2 act upstream in nuclear siRNA biogenesis, producing dsRNAs for processing by DCL3 into 24 nt siRNAs. However, such substrates can also be processed by DCL4 into 21 nt siRNAs, which can act in systemic silencing (Gasciolli *et al.*, 2005; Henderson *et al.*, 2006; Smith *et al.*, 2007; Panda *et al.*, 2020) (Fig. 2F). *dcl3* and *dcl4* mutants in plants expressing a *PHYTOENE DESATURASE* (*PDS*) inverted repeat under the control of the phloem-specific *SUC2* promoter (called the *SUC-PDS* reporter) showed increased and decreased *PDS* gene silencing, respectively. This suggests that mobile silencing requires the 21 nt siRNAs generated by DCL4 from *PDS* dsRNAs rather than the 24 nt siRNAs generated by DCL3. Consistent with this hypothesis, *PDS* silencing was strongly reduced in *dcl3 dcl4* double mutants compared with the *dcl3* single mutant (Smith *et al.*, 2007). Moreover, DCL3-dependent *SUL* siRNAs were neither necessary nor sufficient to mediate cell-to-cell RNA interference in *SUC-SUL* plants expressing a *SUC2* promoter-driven hairpin construct targeting the *SULPHUR* (*SUL*) mRNA (Dunoyer *et al.*, 2007). While these results demonstrated that cell-to-cell RNA silencing relies on 21 nt siRNAs generated by DCL4 (Dunoyer *et al.*, 2007; Smith *et al.*, 2007), long-distance silencing requires signal amplification by RDR6 too (Himber *et al.*, 2003; Brosnan *et al.*, 2007). The role of DCL4-generated 21 nt siRNAs in systemic silencing was confirmed by restoring silencing in *dcl4* mutants with local DCL4 expression in companion cells. Further supporting this view, systemic silencing was blocked by companion cell-specific expression of tombusvirus VSR P19 (Dunoyer *et al.*, 2010), which binds 21 nt siRNA duplexes (Silhavy *et al.*, 2002; Vargason *et al.*, 2003). In other studies, DCL2 was found to be critical for systemic post-transcriptional silencing, highlighting the role of 22 nt siRNAs in initiating transitive silencing required for systemic spread (Mlotshwa *et al.*, 2008; Parent *et al.*, 2015; Taochy *et al.*, 2017).

However, the previous approaches did not clearly distinguish the genetic requirements for systemic silencing between cells producing or receiving the silencing signal. To address this, GFP-expressing scions were grafted on to rootstocks expressing a hairpin construct that produces siRNAs homologous to *GFP* target RNA. This approach confirmed that the spread of transgene silencing is independent of DCL3 expression in the rootstock, which is consistent with the role of 21 nt siRNAs rather than 24 nt siRNAs in transgene silencing signaling. However, Pol IV, RDR2, AGO4, and DCL3 were found to be essential for signal perception and *GFP* mRNA silencing in the scion (Brosnan *et al.*, 2007). Notably, following the grafting of GFP-expressing scions on to rootstocks that produce siRNAs homologous to the *GF* region of the *GFP* target, only siRNAs derived from the *P* region of the *GFP* target were observed in the scion. The sequencing and mapping of *P* region-homologous siRNAs revealed that the siRNAs that mapped to the 5′ part of the *P* region were 24 nt siRNAs, whereas those mapping more distally were 21 nt siRNAs. This finding led to the suggestion that the 24 nt siRNAs guide the synthesis of the 21 nt siRNAs and that, therefore, a Pol IV–RDR2–DCL3–AGO pathway precedes the RDR6–DCL4 production of siRNAs in the recipient cells (Brosnan *et al.*, 2007). However, given that the observation of GFP silencing in the signal-perceiving scion likely also depends on new cell-to-cell silencing generated in the scion tissue after the initial perception of the systemic signal, it is unclear whether the identified genetic components required for GFP silencing in the scion are involved in signal perception, synthesis of new cell-to-cell signal, or both. The notion that small RNAs are mobile and also serve as silencing signals in the absence of transgenic silencing markers is substantiated by the physical presence of both siRNAs and miRNAs in phloem exudates from a range of plant species (Yoo *et al.*, 2004; Buhtz *et al.*, 2008; Varkonyi-Gasic *et al.*, 2010; Rodriguez-Medina *et al.*, 2011). Direct evidence for small RNA movement was provided by small RNA sequencing experiments, which detected the movement of siRNAs from wild-type Arabidopsis scions into the roots of siRNA-deficient Arabidopsis *dcl2 dcl3 dcl4* triple mutants used as the rootstock in grafting experiments (Molnar *et al.*, 2010; Devers *et al.*, 2023). Moreover, *in situ* hybridization and other approaches demonstrated that *TAS3*-derived tasiRNAs expressed in cells at the abaxial side of leaves form a gradient toward the adaxial side, thereby shaping the expression pattern of the target mRNA (Chitwood *et al.*, 2009). Recent studies indicated that the distance of cell-to-cell and systemic movement achieved by small RNAs is contingent upon the involvement of AGO proteins that bind them along the way. Therefore, the distance of small RNA movement is primarily determined by the quantity of small RNAs, their transport between cells, and their consumption by the cell-autonomous silencing machinery in silencing-emitting, traversed, and recipient cells (Devers *et al.*, 2020, 2023; Voinnet, 2022).

In contrast to siRNAs, miRNAs are mostly believed to function in a cell-autonomous manner, because their transcription and activity patterns frequently mirror one another (Parizotto *et al.*, 2004; Alvarez *et al.*, 2006). Nevertheless, while some miRNAs act cell-autonomously (e.g. miR171; Parizotto *et al.*, 2004), a number of miRNAs have been shown to be mobile. For example, the movement of miR165/miR166 is involved in the regulation of cell patterning (McConnell *et al.*, 2001; Nogueira *et al.*, 2007; Carlsbecker *et al.*, 2010). Other miRNAs, such as miR399 and miR395 in Arabidopsis and miR2111 in *Lotus japonicus*, move systemically and are involved in stress responses (Lin *et al.*, 2008; Pant *et al.*, 2008; Buhtz *et al.*, 2010; Tsikou *et al.*, 2018). For instance, miR399 regulates the systemic response to phosphate starvation in Arabidopsis (Fujii *et al.*, 2005), rapeseed, and pumpkin (Pant *et al.*, 2008), while miR2111 regulates lateral root initiation in *L. japonicus* (Sexauer *et al.*, 2023). Using a type of miRNA–GFP sensor system, it has been found that miRNA mobility is spatiotemporally regulated by polarly localized ‘gatekeepers’ that locally regulate miRNA mobility and restrict the spread of miRNAs to specific tissue domains. Thus, miRNAs can be considered as mobile yet discrete signals that behave differently in comparison to proteins and other types of small RNAs that move more freely (Skopelitis *et al.*, 2018).

## Role of plasmodesmata in small RNA mobility

The pathway that allows mobile siRNAs to spread between cells and systemically most likely involves the symplasmic route through the PD. This explains how small RNAs synthesized inside cells can be found in the phloem. Consistent with the movement of small RNAs through PD, mobile silencing signaling was enhanced in the presence of viral movement proteins that modulate the PD aperture (Foster *et al.*, 2002; Vogler *et al.*, 2008) and excluded from spreading into symplastically isolated guard cells (Voinnet *et al.*, 1998). In addition, elegant studies in Arabidopsis embryos showed that the spread of the silencing signal is controlled by diffusion and regulated by the PD aperture (Kobayashi and Zambryski, 2007). The role of PD in silencing spread was further demonstrated in Arabidopsis iCals3m lines, in which CALLOSE SYNTHASE 3 (CALS3)-induced callose deposition at the PD blocked miR156/166 movement (Vaten *et al.*, 2011). Furthermore, inducing CALS3 expression in companion cells retained *SUC-SUL* siRNAs in source leaves and prevented *SUL* silencing in sink leaves (Devers *et al.*, 2023). However, direct evidence that visually demonstrates the movement of small RNAs through the PD is lacking. The observation that miRNA movement can be restricted at local cell–cell interfaces that allow protein movement indicated that miRNA movement is regulated independently of general PD permeability (Skopelitis *et al.*, 2018). While this finding does not rule out a role for PD as conduits for miRNA movement, it may suggest that miRNA movement occurs through an alternative pathway.

## Mobility of small RNAs involved in the regulation of virus infection and symptoms

As explained above, virus-derived siRNAs, mainly 21 nt and 22 nt in length, accumulate during viral infection and are the predominant siRNA class in infected cells. These siRNAs, generated by DCL4 and DCL2 (Fig. 2A) and amplified by RDR6, can move between cells (most likely through PD), presumably to immunize neighboring cells and limit virus spread. For instance, without its P19 VSR, cymbidium ringspot virus replicates in vascular bundle cells but does not spread to surrounding cells (Havelda *et al.*, 2003), and movement of VSR (P38)-deficient turnip crinkle virus was restored in *dcl4* mutant plants lacking 21 nt siRNAs (Deleris *et al.*, 2006). Nevertheless, the efficacy of mobile viral siRNAs in antiviral defense may be mitigated by dynamic changes in the amount and mobility of other siRNA classes in plants. The MP of TMV was shown to enhance the spread of silencing signals, suggesting that siRNAs might also play a proviral role (Vogler *et al.*, 2008). Evidence shows that virus-derived and host-derived siRNAs (e.g. vasiRNAs) can target host gene expression post-transcriptionally (Llave, 2010; Shimura *et al.*, 2011; Smith *et al.*, 2011; Miozzi *et al.*, 2013; Cao *et al.*, 2014; Pitzalis *et al.*, 2020; Leonetti *et al.*, 2021). Thus, in addition to antiviral defense using vsiRNAs, small RNA movement between infected and non-infected cells may include virus- and host-derived siRNAs that target genes and modulate the host environment for the virus.

**Fig. 3.**
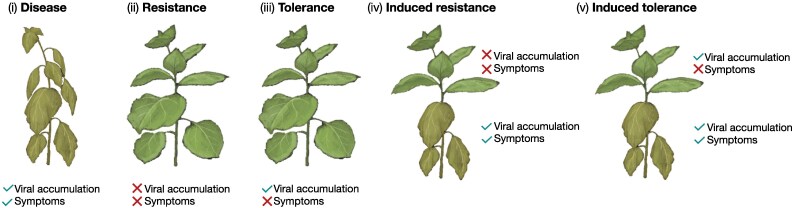
Outcomes of virus infection in susceptible plants. Virus infection can cause disease (i) or plants can remain symptom-free through either resistance (ii) or tolerance (iii). Whereas resistance involves plant defense responses against the virus and results in lower virus titers, tolerance is mediated by plant defense responses against the adverse effects of virus infection. In contrast to resistance, tolerance allows virus titers to be maintained without compromising host fitness. Infection can also trigger induced resistance (iv) or induced tolerance (v), in which plant–virus interactions in symptomatic plants lead to resistance or tolerance only at later stages, resulting in symptom recovery.

Such mechanisms that modulate the host environment for the virus directly affect virus–host compatibility. Although changes in the host environment can have negative effects on virus or host fitness, leading to resistance or disease, respectively, evolution may have guided these plant–virus interactions toward optimization, that is, using the small RNA-induced changes to balance viral and host plant fitness during infection, thereby promoting mutual survival at minimal fitness cost. Because viruses are widespread, such a balance, in which small RNA activity buffers virus accumulation and host gene expression, may be common in natural ecosystems, where virus-infected wild plants are often asymptomatic (Roossinck, 2014, 2015). Such tolerant plants can maintain viral loads without significant effects on growth or reproduction (Paudel and Sanfacon, 2018) (Fig. 3).

Diseases, on the other hand, often arise when a new host or virus is introduced, or when environmental factors disrupt resistance or tolerance mechanisms. Symptoms may result from virus-induced changes in host membranes and cytoskeletal structures, or gene expression, or by virus-induced stress responses and overactive host defenses that divert energy from growth. Viral proteins, particularly VSRs, can play a key role in pathogenesis as they have potential to interfere with host RNA silencing by sequestration of miRNAs (Kasschau *et al.*, 2003; Chapman *et al.*, 2004; Lakatos *et al.*, 2006) or by affecting DCL and AGO proteins (Csorba *et al.*, 2015), all of which can impact plant development. VSRs can also cause symptoms by triggering host immune responses (i.e. ETI), which reduces host fitness and can lead to cell death (Erickson *et al.*, 1999; Li *et al.*, 1999; Malcuit *et al.*, 1999; de Ronde *et al.*, 2014; Wang *et al.*, 2015). Consistent with the major impact of VSRs on viral pathogenicity, their ectopic expression in transgenic plants mimics viral infection symptoms (Kasschau *et al.*, 2003; Chapman *et al.*, 2004; Zhang *et al.*, 2006; Bortolamiol *et al.*, 2007; Malpica-Lopez *et al.*, 2017), whereas VSR mutations reduce symptoms (Chu *et al.*, 2000; Silhavy *et al.*, 2002; Omarov *et al.*, 2006; Vogler *et al.*, 2007; Lewsey *et al.*, 2009; Wu *et al.*, 2010; Malpica-Lopez *et al.*, 2017). However, the mechanisms by which compatible plant–virus interactions control VSR activity to avoid disease are not known.

An excellent opportunity to study these mechanisms is provided by symptom recovery, that is, when plants that initially show symptoms of disease develop healthy, asymptomatic leaves at later stages of development. Recovered leaves often have lower viral RNA levels and are resistant to secondary infections by the same virus but are susceptible to sequence-unrelated viruses (Lindbo *et al.,* 1993; Ratcliff *et al.*, 1999; Siddiqui *et al.*, 2008; Santovito *et al.*, 2014). In plants infected with nepoviruses, the overexpression of a strong VSR, such as potyvirus HC-Pro, prevents recovery and increases virus accumulation (Siddiqui *et al.*, 2008; Santovito *et al.*, 2014). These observations suggest that recovery occurs in plants infected with viruses that have weak VSRs and cannot effectively suppress RNA silencing (Paudel and Sanfacon, 2018). They also show that evolved virus–host interactions may have optimized mechanisms to control VSR activity during infection and thus prevent disease.

However, although antiviral RNA silencing typically lowers virus levels and explains symptom recovery, viral RNA reduction is not always observed in recovered leaves. For instance, in *Nicotiana benthamiana* plants infected with tomato ringspot virus, recovery occurred without changes in viral RNA (Jovel *et al.*, 2007) but was linked to siRNA-mediated translational inhibition (Ghoshal and Sanfacon, 2014). In ORMV-infected Arabidopsis, recovery occurred without affecting viral replication and protein expression, suggesting that recovery in this case was associated with the achievement of a virus-tolerant state. As can be expected, this ‘induced tolerance’ was associated with inhibition of VSR activity, as seen in transgenic plants expressing a silenced *GFP* gene. Whereas GFP silencing was suppressed in the symptomatic leaves, indicating VSR activity, GFP was re-silenced in recovered leaves despite continued VSR expression, thus indicating inactivation of the protein (Kørner *et al.*, 2018).

**Fig. 4.**
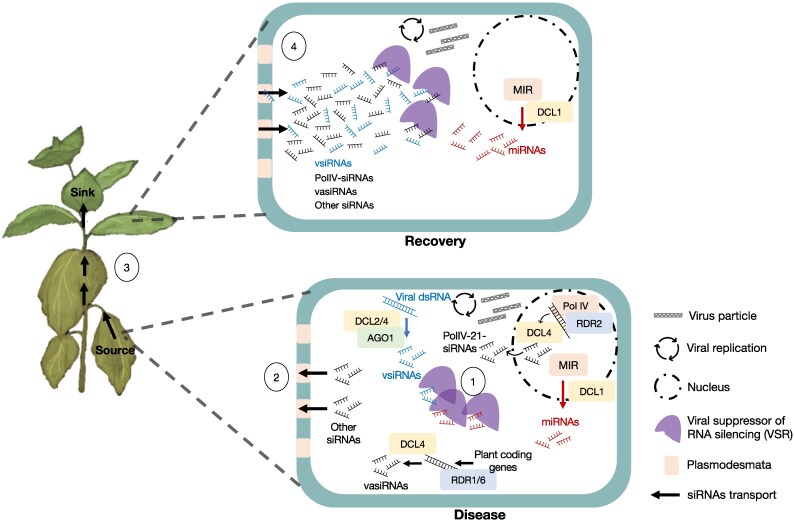
Hypothetical model of siRNA-mediated induced tolerance leading to symptom recovery. (1) In highly infected carbon source leaves expressing high levels of VSR, VSR sequesters both miRNAs and vsiRNAs. This disrupts miRNA function in gene regulation and leads to disease. At the same time, viral infection induces the production of viral and host-derived 21 nt siRNAs through the RDR1/6-DCL4 pathway (including vasiRNAs) and non-canonical processing of Pol IV–RDR2 products by DCL4 (Pol IV-21-siRNAs). (2) These siRNAs move from cell to cell through the plasmodesmata and (3) systemically through the phloem to reach the young carbon sink tissues ahead of the virus. (4) In the young sink tissues, the accumulating siRNAs saturate the small RNA binding activity of the VSR of the incoming virus, thereby reducing its disruption of miRNA pathways. As a result, disease induction is prevented, resulting in symptom recovery at the plant phenotypic level. The virus-induced production of mobile siRNAs and the ability of these siRNAs to control VSR activity drives the transition to a state of induced tolerance while the virus continues to replicate and produce virus particles.

Genetic analysis of symptom recovery in ORMV-infected Arabidopsis revealed that the process of induced tolerance depends on components of cytoplasmic and nuclear siRNA synthesis (DCL4, SGS3, RDR6, Pol IV, RDR2), methylation (HEN1), and AGO1, but not on miRNA synthesis or DNA methylation and chromatin-remodeling proteins (Kørner *et al.*, 2018). Recovery was enhanced in *dcl3* mutants, suggesting the involvement of 21 nt siRNAs derived from DCL4 processing of Pol IV–RDR2 products, which are also involved in silencing signaling (Dunoyer *et al.*, 2007; Smith *et al.*, 2007; Kørner *et al.*, 2018) (Fig. 2F). Enhanced recovery was also observed in *xrn4* mutants, which, in the absence of 5′–3′ RNA decay mediated by XRN4, may provide increased levels of RNA substrates for RDR6-mediated siRNA amplification (Moreno *et al.*, 2013; Li and Wang, 2018) (Fig. 2A) or convert RNA decay substrates into ct-siRNAs (Gazzani *et al.*, 2004; Moreno *et al.*, 2013; Zhang *et al.*, 2015; Feng *et al.*, 2024) (Fig. 2E). Overall, the findings suggested that reaching a tolerant state in ORMV-infected Arabidopsis plants may depend on cytoplasmic 21 nt vsiRNAs generated from DCL4 processing of viral dsRNA, cytoplasmic vasiRNAs generated by RDR6 and DCL4 from host transcripts, and 21 nt siRNAs generated by DCL4 processing of nuclear Pol IV–RDR2 products (PolIV-21-siRNAs) (Fig. 4). Symptom recovery and VSR inactivation occurred in sink tissues, which led to the proposal that these siRNAs from symptomatic source tissues compete with miRNA binding by the VSR in sink tissues, thereby preventing symptoms (Fig. 4) (Kørner *et al.*, 2018). This suggests that intercellular communication and virus-induced siRNA synthesis and systemic mobility play a role in buffering VSR activity and thus preventing disease through induced tolerance. Further studies are needed to determine the exact mechanism. Although the mobile siRNAs may occupy the small RNA binding sites of the VSR, thus preventing its interference with miRNA-mediated gene regulation, the VSR may also be regulated by post-translational modifications. Moreover, as already mentioned, virus-induced siRNAs such as vsiRNAs and vasiRNAs have the potential to target host genes for post-transcriptional silencing (Pantaleo *et al.*, 2007; Qi *et al.*, 2009; Shimura *et al.*, 2011; Smith *et al.*, 2011; Cao *et al.*, 2014; Pitzalis *et al.*, 2020; Leonetti *et al.*, 2021). Therefore, their systemic mobility during virus infection may regulate the outcome of infection in newly infected leaves not only by regulating the VSR, but also by regulating host genes.

## Induced tolerance as a model to study tolerance

Tolerance, like resistance, is a defense response. However, whereas resistance acts against the virus, tolerance acts against the negative impact of infection. Thus, unlike resistance, tolerance allows the plant to maintain a high viral load without consequences on fitness. The above-mentioned study on induced tolerance (Kørner *et al.*, 2018) showed that tolerance involves RNA silencing pathways and confirms that tolerance and recovery occur without affecting virus levels, indicating that disease symptoms are not always linked to virus accumulation. This observation highlights how tolerant virus–host interactions balance fitness costs, allowing the plant and virus to coexist without extreme costs. Induced tolerance in ORMV-infected Arabidopsis plants offers a model to further study the molecular basis of tolerance, which is common in nature (Roossinck, 2015). Mobile siRNAs involved in induced tolerance may also play a key role in natural tolerance, and identifying genes encoding these siRNAs could help to breed virus-tolerant crops. Thus, tolerance offers the potential for plant disease control and insights into plant–pathogen interactions in wild populations (Pagan and Garcia-Arenal, 2020).

## Conclusion

Virus-infected plant cells exhibit altered small RNA profiles, with key changes in plant miRNA levels and in the accumulation of virus- and host-genome-derived siRNAs. These siRNAs can move from cell to cell and systemically in the vasculature to exert control over virus accumulation and gene expression, contributing to virus–host compatibility and disease control. As discussed in this review, disease is often associated with highly expressed VSR proteins that disrupt plant development by sequestering miRNAs. Plants can control disease through resistance, in which both virus and VSR levels are kept low, or through tolerance, in which the virus and VSR levels remain high but disease is managed. Based on analyses of induced tolerance, we propose that tolerance suppresses disease through mechanisms that control VSR activity, where the virus- and host-derived siRNAs occupy small RNA binding sites of the VSR, preventing miRNA sequestration and symptom development. In plants recovering through induced tolerance, this infection-induced siRNA pool moves from infected source leaves to sink tissues, where it prevents disease (Fig. 4).

The movement of siRNAs out of initially infected cells is a key process likely to influence induced tolerance and conventional tolerance. This siRNA movement is regulated by the PD size exclusion limit at the infection front, that is, by the interplay between host PTI, which aims to close the PD, and viral MP, which acts to prevent this closure. Further studies are needed to clarify whether induced tolerance results from the siRNA-mediated inhibition of miRNA sequestration by the VSR, from systemic siRNA control of host genes, or from a combination of both. Nevertheless, the identification of plant genes encoding virus-induced siRNAs could aid in the breeding of virus-tolerant plants, providing a more stable plant protection strategy than resistance (Pagan and Garcia-Arenal, 2018, 2020). Although tobamoviruses were thought to have a narrow host range (Gibbs *et al.*, 2015), they have since been found to infect diverse species (Zamfir *et al.*, 2023). Therefore, the tolerance mechanisms identified using tobamoviruses in Arabidopsis may apply to many wild plants and pathosystems. Understanding tolerance could provide insights into viral communities, plant–virus ecology, and the effects of environmental factors and climate change.
